# Metrology of frequency comb sources: assessing the coherence, from multimode to mode-locked operation

**DOI:** 10.1515/nanoph-2023-0805

**Published:** 2024-03-13

**Authors:** Roberto Eramo, Alessia Sorgi, Tecla Gabbrielli, Giacomo Insero, Francesco Cappelli, Luigi Consolino, Paolo De Natale

**Affiliations:** 68229 CNR-INO – Istituto Nazionale di Ottica, Via Carrara, 1 – 50019, Sesto Fiorentino FI, Italy; LENS – European Laboratory for Non-Linear Spectroscopy, Via Carrara, 1 – 50019, Sesto Fiorentino FI, Italy; Department of Experimental and Clinical Biomedical Sciences “Mario Serio”, 9300 University of Florence, Viale Pieraccini, 6 – 50139, Firenze FI, Italy

**Keywords:** frequency comb, coherence, mode locking, laser

## Abstract

Since the beginning of this millennium, frequency comb generators have reshaped frequency metrology and related areas. After more than two decades since their first realization, several other ways to generate frequency combs, in any spectral region, have been demonstrated, each way with its peculiar features. This trend has triggered the need to quantitatively assess how close the new comb realizations are to an ideal comb, a feature that will be called combness throughout this paper. We will briefly review the very dynamic area of novel frequency comb sources and we will describe the techniques that have been recently developed to quantitatively assess the key parameters of old and new frequency combs, in view of the specific applications. Finally, we will try to sketch future steps in this recently born research area.

## 1 Introduction

For many years, at the end of the previous century, the development of ultrafast laser sources did not cross frequency metrology that strived to reference optical frequencies to the Cesium clock transition in the microwaves, at 9.19 GHz, chosen as primary frequency standard. To this purpose, frequency chains including plenty of phase-referenced ultra-stable continuous-wave lasers had been developed. Then, across the year 2000, at the beginning of this millennium, the intrinsic correlation in between time and frequency domains was finally unveiled and exploited: the frequency comb (FC) generator was born. This new concept appeared to be groundbreaking not only for frequency metrology and plenty of related areas, but also for new concepts of laser sources, designed to generate “combs” in a number of different ways and in widely different spectral regions.

### 1.1 The evolution and diversification of optical frequency comb synthesizers

FCs are nowadays key tools in many fields of fundamental and applied research [1]. Visible/near-infrared FCs can be generated by means of controlled, frequency-stabilized mode-locked femtosecond lasers [2], [3], [4]. In recent years, FCs have been rapidly developed on fiber-based platforms, achieving a high operation power and stability, and broad spectral coverage in the visible and near-infrared spectral regions.

The miniaturization of the sources, together with the expansion of their operation towards other spectral regions (ultraviolet, mid- and far-infrared), is crucial for broadening their application range (telecommunications [5], [6], molecular spectroscopy and gas sensing [7]).

Specifically, the availability of broadband and compact radiation sources, spectrally covering the ultraviolet range while exhibiting optical phase coherence and high repetition rates opens the doors to precision frequency metrology, photoelectron spectroscopy and attosecond science [8], [9], [10], [11], [12], [13]. In the absence of convenient broadband laser gain media and optics suitable for building mode-locked oscillators in the ultraviolet range, frequency upconversion of the pulse train emitted by visible/near-infrared mode-locked lasers via coherent high-order harmonic generation is the only available approach at the moment [14].

On the other side of the electromagnetic spectrum, the generation of mid-to-far infrared FCs is possible via two different approaches. The first one consists in down-converting near-infrared FCs via difference-frequency generation (DFG) [15], [16], [17], [18], [19] or using synchronously-pumped optical parametric oscillators (OPOs) [20], [21]. Conceptually, a DFG-based mid-infrared FC (DFG-comb) is obtained by mixing a visible/near-infrared FC with a pump laser in a non-linear crystal, with matching wavelength requirement to reach the mid infrared [22], [23], [24], [25]. At even longer wavelengths, the generation of a THz FC via optical rectification has been demonstrated [26].

On the miniaturization front, the most interesting results have recently been achieved with the following technologies: microresonators, quantum cascade lasers and interband cascade lasers. All of them are characterized by a high third-order (Kerr) nonlinearity and by the same FC formation mechanism, that is degenerate and non-degenerate four-wave mixing (FWM).

Whispering gallery mode resonators (microresonators) are ring microcavities with large *Q* factors $\left(∼10^{8}\right)$ on a broad spectral range [27], [28], [29], [30]. Pumped with continuous-wave lasers, thanks to the nonlinear effects taking place within the waveguide, they are able to generate FCs [31] in the visible/near-IR regions. The variety of emission states microresonators exhibit is very rich, spanning from chaotic emission to coherent multimode emission and even short-pulses emission in the temporal dissipative solitons regime [32], [33]. The operating regime strongly depends on the specific microresonator optical properties (in particular on dispersion) and on the pumping optical power and frequency mismatch with respect to the closest microcavity resonance. An intriguing and recently-emerged alternative relying on second order non-linear processes consists in the generation of FCs starting from continuous-wave single-mode lasers by exploiting quadratic cascading generation in non-linear crystals [34].

On the other hand, quantum cascade lasers (QCLs) are current-driven semiconductor lasers based on intersubband transitions in quantum wells, emitting high-power coherent radiation in the mid and far infrared [35], [36], [37]. Due to the active region structure, in particular for high-performance room-temperature mid-infrared devices, the upper lasing state lifetime is very short compared to the cavity round-trip time (about two orders of magnitude). As a consequence, in continuous-wave operation, energy cannot be stored during the round trip, the sustenance of optical pulses is prevented and classical pulsed passive mode locking is generally not achievable [38], [39], [40]. In this regard, new results related to the implementation of graphene-based saturable absorbers were recently demonstrated [41].

Active mode locking emerged as an alternative to passive mode locking. Active pulsed mode locking has been successfully demonstrated both in the mid infrared [42], [43] and THz [44], [45] ranges. The limitation of this approach derives from the need of close-to-threshold operation in order to mitigate gain saturation, severely limiting the emitted power, and from the length of the pulses that cannot reach the inverse of the gain bandwidth.

However, by using broadband Fabry–Pérot QCLs [46], [47] designed to have low group velocity dispersion, FC generation has been demonstrated in free-running operation (QCL-combs) both in the mid-infrared and in the THz range [38], [48], [49]. Starting from the independent longitudinal modes generated by a Fabry–Pérot multimode laser, degenerate and non-degenerate FWM processes induce a proliferation of modes over the laser emission spectrum [50], [51]. The original modes are then injection-locked by the modes generated by FWM, ensuring correlation among all the longitudinal modes, giving birth to a FC [40] with a fixed phase relation, but a non-pulsed emission. In the THz range the full phase stabilization of both the FC degrees of freedom (offset and mode spacing) has been demonstrated [52], while FC operation over the entire available gain bandwidth has been achieved by conveniently increasing the mirror losses of the Fabry–Perot cavity through coating the back facet with an epitaxially-grown multilayer graphene film [53].

More recently, interband cascade lasers (ICLs) [54], [55], [56], [57], [58] also proved to be able to generate FCs [59], [60], [61]. Both QCLs and ICLs could be successfully exploited for dual-comb spectroscopy (DCS) [62], [63], [64] and free-space communication [65], [66].

Advanced techniques for FC characterization have been developed. Frequency-resolved optical gating (FROG) [33], [67], spectral phase interferometry for direct electric-field reconstruction (SPIDER) [68], [69] and asynchronous upconversion sampling (ASUPS) [70] have been developed and applied to characterize FC emission in the time domain. Other techniques, suitable for studying quasi-continuous-wave mid- and far-infrared radiation, have been developed. The list comprises optical and RF spectrum monitoring, intermodal beatnote spectroscopy [38] and shifted wave interference Fourier transform spectroscopy (SWIFTS) [48], single-frequency counting and multi-heterodyne detection using a dual-comb setup for frequency equispacing estimation and frequency stability characterization [62], [71], the Vernier technique using a high-finesse optical cavity for technical and quantum frequency noise estimation [72]. All these techniques afford coherence estimation of the FC emission. Among them, only the SWIFTS technique can access the phase relation between the modes [73], [74], [75]. In particular, it allows for the retrieval of the phase of each mode compared to the ones of its first neighbors. As a consequence, this technique enables to obtain the phase relation inherent to continuous portions of the FC spectrum. The main limitation is that SWIFTS relies on a cumulative sum, therefore the result is particularly subject to noise. Moreover, relying on a scan (in particular the mechanical scan of the interferometer arm, usually lasting 5–15 min [48]) it does not allow for a synchronous retrieval of all the modal phases, preventing monitoring of the time evolution of the phase relation.

In 2019 our research group proposed an alternative characterization technique named Fourier-transform analysis of comb emission (FACE). The experimental procedure, firstly reported in ref. [76] and then deeply discussed in ref. [77], takes advantage of the multiheterodyne detection scheme, also used in dual-comb spectroscopy setups, the major difference being that the sample to be investigated is not a molecular species but the sample FC itself. Following this scheme, the sample FC is mixed with a second, fully controlled and stabilized, reference FC (LO), generating a down-converted FC in the radio frequencies (RF) domain. The phase information, encoded in the RF beat notes, is then retrieved by means of a subsequent Fourier-transform analysis. The main advantages of the FACE technique are its great generality, i.e. its applicability to any FC source, regardless of its wavelength or temporal waveform, and its remarkably simple experimental setup, in which only a fast mixer and a reference FC (spectrally overlapping with the sample one) are needed for the down-conversion. Another important aspect is that, unlike SWIFT, FROG, and SPIDER, the FACE technique (being based on DCS) does not require a mechanical scanning arm, and can therefore provide a simultaneous and real-time sampling of the investigated FC.

In this work, a mid-infrared QCL-comb is investigated as sample, its spectral coherence is evaluated showing how the coherence time of each FC mode can be retrieved. This parameter is proposed as estimator for the *combness*.

## 2 Discussion

### 2.1 Evaluating the coherence of frequency comb modes

We will illustrate the approach of the Fourier-transform analysis of comb emission (FACE) considering the steps necessary for deriving a coherence plot of a mid-infrared laser; we refer for the details to ref. [77]. In a nutshell, FACE is a dual-comb spectroscopy (DCS) without spectroscopic sample, where a *local oscillator* FC (LO-FC, repetition frequency $\nu_{\text{rep}}^{\left(\text{lo}\right)}$ ), usually a passive mode-locked pulsed FC, is fully known, meaning in the simplest case that all modes involved in the beating have the same (complex) amplitude. On the other side, the *sample FC* (S-FC, with repetition frequency $\nu_{\text{rep}}^{\left(\text{s}\right)}$ ) is the FC under investigation. The S-FC ad LO-FC are interferometrically overlapped, and then sent to a detector in order to generate a heterodyne beat signal, digitally acquired as a complex IQ waveform with a real-time spectrum analyzer.1For few details about the IQ waveform, see the signal scheme reported in ref. [77]. Only the heterodyne difference frequency terms are of interest, as they give rise to nearly monochromatic beat oscillators when the relative phase of the two FCs is kept constant.

**Figure 1:**
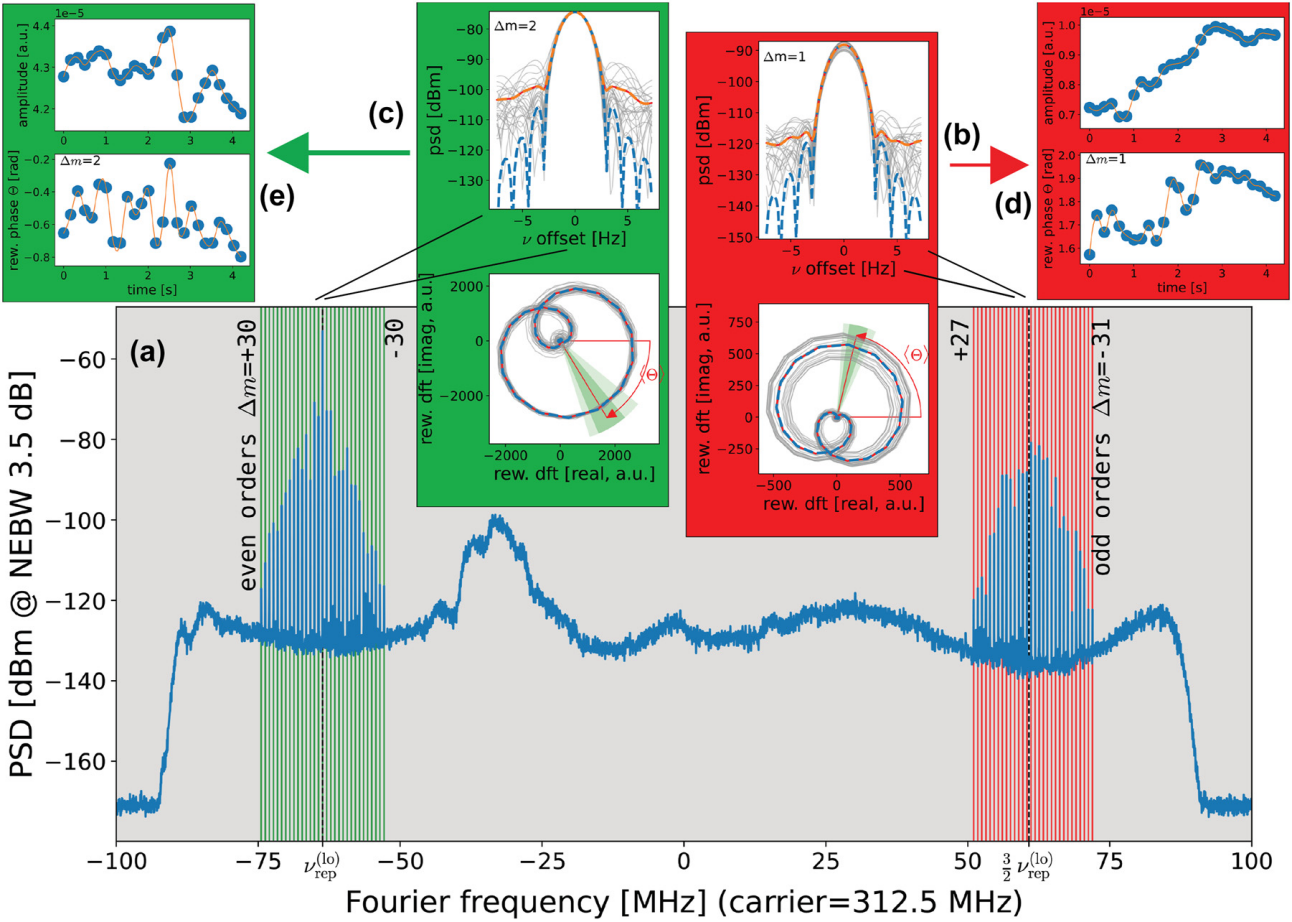
Example of FACE data analysis in the case of a *semi-harmonic mixing* condition. The IQ-data from the spectrum analyzer is split in strides (duration *T*_*s*_ = 0.67 s) and each stride is analyzed through a FFT routine. (a) PSD showing the underlying FC structure: the mode Δ*m* = 0 corresponds to the RBN at frequency $\nu_{\text{rep}}^{\left(\text{lo}\right)}$ ; around it we find the FC of the even order modes, while around $\frac{3}{2}\nu_{\text{rep}}^{\left(\text{lo}\right)}$ we find the FC of the odd order modes. The plot resolution is increased around each nominal mode frequency (vertical lines in green and red) to render properly the narrow peaks. Insets (b) and (c): in the top part a detail of the average PSD, and the PSD of each stride (light-gray); in the bottom part the DFT is plotted on the complex plane, showing the characteristic shape of the adopted window function, whose axis angle gives the measured stride phase (Θ). Also for the complex plot we show the average value of the DFT and the DFT of each stride (in light gray). The shaded angular region indicates the standard deviation of the oscillator phase (rewound at *t* = 0). The frequency resolution of these plots ( $∼2^{3}$ samples in the Fourier width 1/*T*_*s*_) is determined by the zero padding of the original IQ waveform. (d) and (e) Oscillator amplitude (top) and phase (bottom) versus stride time, as derived from (b) and (c).

Beside the simplest case, when the repetition frequencies of the two FCs are just slightly detuned of a quantity Δ*ν*_rep_, $\nu_{\text{rep}}^{\left(\text{s}\right)}=\nu_{\text{rep}}^{\left(\text{lo}\right)}+\Delta \nu_{\text{rep}}$ , which is the condition of traditional DCS; the structure of the heterodyne signal is reasonably simple also in other conditions. We discussed in our previous works the condition of *harmonic mixing*, when there is a nearly matching between the repetition rate of one of the two FCs and a harmonic of the other, e.g.

(1) $$\nu_{\text{rep}}^{\left(\text{s}\right)}=k\nu_{\text{rep}}^{\left(\text{lo}\right)}+\Delta \nu_{\text{rep}}$$

where *k* is an integer number defining the harmonic mixing ratio. In this work, we will consider a case of *semi-harmonic mixing*, when $\nu_{\text{rep}}^{\left(\text{s}\right)}=\left(k+\frac{1}{2}\right)\nu_{\text{rep}}^{\left(\text{lo}\right)}+\Delta \nu_{\text{rep}}$ ; this case is of interest when, due to experimental constraints (e.g. the case study discussed below), the harmonic mixing condition cannot be achieved. It can be shown that in this case, with our standard processing of the heterodyne signal, which has the purpose of subtracting the RF oscillators residual phase noise common to a selected *reference beat note* (RBN), the even order beat notes (i.e. with even offset index Δ*m* respect to the RBN) are RF FCs around the frequencies $q\nu_{\text{rep}}^{\left(\text{lo}\right)}$ , while odd order beat notes are RF FCs around $\left[q+\frac{1}{2}\right]\nu_{\text{rep}}^{\left(\text{lo}\right)}$ (*q* integer). Each of these RF FCs has a frequency separation 2Δ*ν*_rep_, and can be considered a mapping of the original optical S-FC, in the sense that the amplitude of the RF oscillator ${\text{e}}^{\text{i}\left(q\nu_{\text{rep}}^{\left(\text{lo}\right)}-\Delta m\Delta \nu_{\text{rep}}\right)t}$ (Δ*m* = 0, ±1, …), or of the oscillator ${\text{e}}^{\text{i}\left(\left[q+\frac{1}{2}\right]\nu_{\text{rep}}^{\left(\text{lo}\right)}-\Delta m\Delta \nu_{\text{rep}}\right)t}$ (Δ*m* = ±1, ±3, …), is proportional to a corresponding optical S-FC amplitude. An important experimental requirement is that these even/odd order RF FCs must not overlap. In the case of S-FC with large frequency extension this requires a narrowband optical filter in order to reduce the RF FCs extension. In the example we are going to show, this was not necessary, as the bandwidth of the S-FC is sufficiently limited. Thus, we are able to gain information about amplitude and phase of each S-FC optical mode by analyzing the complex amplitude of the corresponding RF oscillator. In Figure 1, an example of FACE data analysis, in the case of *semi-harmonic mixing*, is reported. The employed LO-FC is a DFG-comb by Menlo Systems [25]. An Yb-doped mode-locked fiber laser emitting pulsed light centered on 1040 nm with a repetition rate of 249 MHz is used as seed. The repetition rate can be tuned and stabilized via a piezoelectric actuator and an electro-optic modulator. The DFG takes place in a 3-mm-long multiperiod periodically-poled lithium niobate (PPLN) crystal. The generated radiation is a pulsed MIR DFG-comb working around a wavelength of 4.4 µm with an average power of up to 300 mW inheriting the repetition rate of the seeding laser. The S-FC is a QCL-comb by ETH Zurich operating around 4.4 µm [75]. At the operating temperature of −8 °C, the FC mode spacing is 10.084 GHz.2Due to the limited tunability of both the DFG-comb repetition rate and the QCL-comb mode spacing, it was not possible to reach a harmonic mixing ratio (see Eq. (1)). The reached semi-harmonic mixing ratio is 40.5. The two beams are superimposed and detected via a fast MCT detector by VIGO Photonics. During the acquisitions, the repetition rate of the LO-FC and the mode spacing of the S-FC are phase locked to two RF oscillators referenced to the primary standard to increase the mutual phase coherence of the two sources. Moreover, a dedicated RF chain takes care of subtracting in the dual-comb multiheterodyne signal one reference beat note from all the others in order to remove the common-mode noise [76]. After the RF chain, the processed signal is acquired with an RF spectrum analyzer as IQ time traces. The IQ data streams are segmented in subsequent *strides* (duration 0.67 s, sampling rate *F*_*s*_ = 1 GHz), multiplied by the window function for tapering purpose (here we choose a Hann window), and for each stride the discrete Fourier transform (DFT) is calculated through a FFT routine. The DFT segment duration is calibrated on the mode oscillators under investigation, as the mode DFT must be compatible with a quasi–monochromatic oscillator (for the given stride duration and window function) in order to be able to extract from it the modal phase and amplitude as a function of the stride time. A factor 2^3^ is adopted for the zero-padding, in order to interpolate the instrumental response (thus we are considering FFT of arrays of 2^3^ × 0.67 G samples). In Figure 1a the power spectral distribution (PSD) is shown, which is proportional to the square modulus of the stride’s DFT, averaged over all the strides. The PSD shows the underlying FC structure of the heterodyne beats discussed before. In Figure 1 we sketch the analysis that can be made on each oscillator, following it for two modes (insets (b) and (c)): in the top part, a detail of the average PSD (orange) around the mode frequency is shown, together with the PSD of each stride (light-gray); in the same figure the instrumental response, that in the PSD context is given by the square modulus of the Fourier transform of the window function, is also shown for comparison (blue). In the bottom part of the inset the DFT is plotted on the complex plane, showing the characteristic *roselet* shape of the Fourier transform of the window function,3The window choice, determining the frequency contribution of the stride selection in the whole data buffer, also determines the analytical shape of the DFT of an ideal oscillator, and can thus be considered the instrumental function of our technique. As compared to our previous work [77], where a simple *boxcar* window was used, here we adopted a classical Hann window. In any case, the mode DFT is given by a proportional factor, including the mode amplitude/phase, times the instrumental function; thus we can easily extract the mode amplitude/phase from the DFT, and they are independent of the window choice. that in the complex plane plays the role of instrumental function, and whose axis angle gives the measured phase oscillator (Θ_Δ*m*_) at the given stride time; for each oscillator the DFT is *rewound* to the time origin, i.e. we removed in Θ_Δ*m*_(*t*) the natural phase evolution factor ${\text{e}}^{2\pi i\nu_{\Delta m}t_{s}}$ calculated for the nominal mode frequency *ν*_Δ*m*_ and stride time *t*_*s*_, in order to compare the different RF oscillators at *t* = 0. Also for the complex plots (Figure 1b and c) we show the average DFT and the DFT of each stride (in light gray). For a complete incoherent oscillator the average would not show any axis, and its shape would not be given by the window function FT. The shaded angular region measures the standard deviation of the oscillator phase. Finally, in Figure 1d and e we plot the oscillator amplitude (top) and phase (bottom) versus stride time, as derived from the analysis sketched in insets (b) and (c).

**Figure 2:**
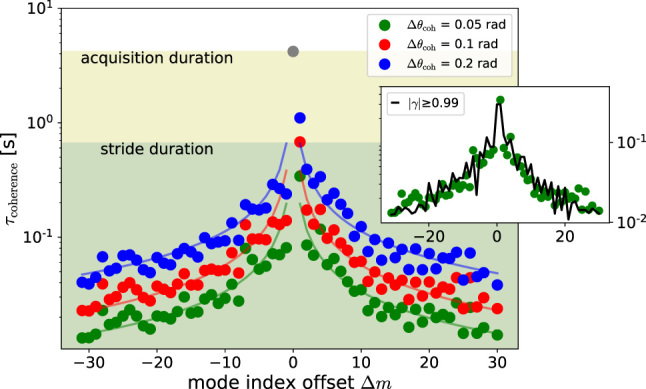
Average coherence time as a function of the mode index Δ*m* for three values of threshold angle Δ*θ*_coh_ (0.05, 0.1, and 0.2 rad). The reference mode Δ*m* = 0, which is taken as phase reference, reaches the full acquisition duration without hitting the threshold, and is plotted as a gray dot. For the other oscillators the coherence time is fitted with a power law *τ* = *A*/|Δ*m*|^*α*^ (Δ*m* = 0 datum excluded). The exponent fit parameter is *α* ≈ 0.8 ± 0.07. In the inset the Δ*θ*_coh_ = 0.05 mrad data is compared with the alternative *γ*–derived modal coherence (here |*γ*| ≥ 0.99), black line, showing essentially the same result.

As a result of this analysis we have the amplitude and the phase of each oscillator in the heterodyne beats as a function of the stride time. The phase knowledge opens the possibility to investigate the time coherence of the oscillators. To this purpose we set three threshold values for the oscillator phase angle (Δ*θ*_coh_ = 0.05, 0.1, and 0.2 rad) and calculate the average time (*τ*_choerence_ in figure), that the rewound phase Θ_Δ*m*_ − Θ_0_ needs in order to drift of the threshold value. The result is shown in Figure 2. The RBN, the phase reference, reaches the full acquisition duration without hitting the threshold, and is plotted as a gray dot. For the other oscillators the coherence time is found of the order or below the stride duration, and as expected, is decreasing for increasing |Δ*m*|. For the three threshold values we found that a power law *τ* = *A*/|Δ*m*|^*α*^ is a good fit for the data (once the Δ*m* = 0 point is excluded). The exponent fit parameter is *α* = 0.77 ± 0.07 for both Δ*θ*_coh_ = 0.05 rad and 0.2 rad, while *α* = 0.83 ± 0.08 for Δ*θ*_coh_ = 0.1 rad. As a further check, we can use the classical expression for the *complex degree of coherence* [78]

$$\gamma_{\Delta m}\left(t,\tau \right)=\frac{\left\langle E_{\Delta m}\left(t+\tau \right)E_{\Delta m}^{*}\left(t\right)\right\rangle }{\sqrt{\left\langle |E_{\Delta m}\left(t+\tau \right){|}^{2}\right\rangle \;\left\langle |E_{\Delta m}\left(t\right){|}^{2}\right\rangle }}$$

where $E_{\Delta m}\left(t\right)=A_{\Delta m}\left(t\right){\text{e}}^{\text{i}\Theta_{\Delta m}}{\text{e}}^{2\pi i\nu_{\Delta m}t}$ is proportional to the Δ*m* mode field, being *A*_Δ*m*_ and Θ_Δ*m*_ the real amplitude and phase that we obtain from our analysis (e.g. insets (e) and (d) in Figure 1). As our mode oscillators do have slowly-varying (complex) amplitudes – they have indeed the stride time as characteristic time scale, much longer than the oscillators period – we can suppose that the average in the *γ* definition just filters out the fast frequency *ν*_Δ*m*_, while is ineffective on the complex amplitudes, giving a degree of coherence only determined by the phase fluctuations:

$$\gamma_{\Delta m}\left(t,\tau \right)\approx {\text{e}}^{2\pi i\nu_{\Delta m}\tau }{\text{e}}^{\text{i}\left[\Theta_{\Delta m}\left(t+\tau \right)-\Theta_{\Delta m}\left(t\right)\right]}$$

Finally, considering ΔΘ_Δ*m*_ ≡ Θ_Δ*m*_(*t* + *τ*) − Θ_Δ*m*_(*t*) as a stationary (*t*–independent) stochastic Gaussian process, we obtain4An inessential phase factor, due to ⟨ΔΘ_Δ*m*_⟩, is neglected in the following expression: we will be interested on the modulus |*γ*|.

(2) $$\begin{array}{ll} \gamma_{\Delta m}\left(\tau \right) & \equiv \left\langle \gamma_{\Delta m}\left(t,\tau \right)\right\rangle \\ & ={\text{e}}^{2\pi i\nu_{\Delta m}\tau }\;\left\langle {\text{e}}^{\text{i}\Delta \Theta_{\Delta m}\left(\tau \right)}\right\rangle \\ & ={\text{e}}^{2\pi i\nu_{\Delta m}\tau }{\text{e}}^{-\frac{1}{2}\left\langle \Delta \Theta {\left(\tau \right)}^{2}\right\rangle } \end{array}$$

where averaging is now made on the process realizations, i.e. over *t* if the further assumption of ergodicity is made, and the last passage stems from a well-known identity of the characteristic function $\left\langle {\text{e}}^{\text{i}\Delta \Theta_{\Delta m}\left(\tau \right)}\right\rangle$ for a process with a normal distribution.5We have checked that indeed for our data the characteristic function average gives the same result of the final expression of *γ*_Δ*m*_(*τ*), which is only based on the phase variance, i.e. that with a good approximation the phase jumps behave as a Gaussian ergodic noise in the investigated range. We are now able to obtain for each mode a *γ*–derived coherence time setting a minimum threshold on |*γ*(*τ*)| (or equivalently a maximum threshold on ⟨ΔΘ(*τ*)^2^⟩).6|*γ*(*τ*)| reaches its maximum (i.e. 1) for *τ* = 0 and decreases monotonically for increasing |*τ*|: we could check this for |*τ*| ≤ 0.5 s. In the inset of Figure 2 the modal *γ*–coherence time (threshold |*γ*| ≥ 0.99) is compared with the previous approach (ΔΘ_coh_ = 0.05 rad) showing essentially the same behavior, and we can conclude that, at least with the current set-up, the two ways of estimating the modal coherence are essentially equivalent.

We conclude observing that this kind of characterization concerns the FC interferometric coherence, as it measures the phase stability of each S-FC mode; the phase stability is also influenced by the phase stabilization electronic loop. Moreover, since it is related to slow phase drifts, it cannot be estimated by the traditional PSD analysis.

## 3 Conclusions

Considering the wide variety of possible frequency comb patterns that can be generated exploiting different physical phenomena, devices and materials, the need to perform quantitative measurements clearly arises in order to assess how close a specific comb is to an ideal frequency comb, a feature that we call *combness*. We have shown that a key parameter to assess the *combness* is the phase stability of each single mode, relative to all the others. After discussing different existing methodologies to make such a characterization, we focus the attention on what we consider the most rigorous technique to this goal, the FACE technique. In combination with a fully-fledged complex field data analysis, FACE allows to determine the actual coherence time of each and every comb mode, providing quantitative data for FC use in experiments/applications with specific targets and requirements.

This perspective work highlights also the need to summarize the many data that are usually collected, to build smart and easy–to–use quantitative *combness* indicators. In addition, smarter ways to get data, reducing the overall data load and measurement time, can help the ranking of existing FCs, in view of a wider and wider range of applications, more or less demanding of the actual FC properties. As always happens when accurate quantitative approaches are set-up, we believe that FC applications will mostly benefit from these new methodologies, as well as future FC generators.
